# Exploring the Role of the TAS2R16 Protein and Its Single Nucleotide Variants in Pituitary Adenoma Development

**DOI:** 10.3390/biomedicines12092022

**Published:** 2024-09-04

**Authors:** Enrika Pileckaite, Alvita Vilkeviciute, Greta Gedvilaite-Vaicechauskiene, Loresa Kriauciuniene, Rasa Liutkeviciene

**Affiliations:** 1 Laboratory of Ophthalmology, Institute of Neuroscience, Lithuanian University of Health Sciences, LT-50161 Kaunas, Lithuania; alvita.vilkeviciute@lsmu.lt (A.V.); greta.gedvilaite@lsmu.lt (G.G.-V.); loresa.kriauciuniene@lsmu.lt (L.K.); rasa.liutkeviciene@lsmu.lt (R.L.); 2 Department of Ophthalmology, Medical Academy, Lithuanian University of Health Sciences, LT-50161 Kaunas, Lithuania

**Keywords:** *TAS2R16*, ELISA, pituitary adenoma, single-nucleotide variants of TAS2R16 gene

## Abstract

Background: Pituitary adenoma (PA) is a common benign tumor that develops in the pituitary gland, causing hormonal imbalances and potential health issues. The *TAS2R16* gene codes for a taste receptor and is involved in bitter taste perception, but there is currently no known direct link between this gene and pituitary adenoma. Methods: This study included 221 healthy controls and 131 patients with pituitary adenoma (PA) from the Lithuanian population. DNA was isolated from peripheral venous blood using the salt precipitation method. Genotyping was performed via RT-PCR. Statistical analysis was conducted with IBM SPSS Statistics 29.0 software, incorporating the Bonferroni correction for multiple comparisons. Results: This study found that the *TAS2R16* rs978739 C allele is less common in the non-invasive PA group compared to the control group (*p* = 0.045). The *TAS2R16* rs860170 CT genotype reduces the likelihood of developing non-invasive PA by 1.9-fold under the codominant (*p* = 0.024) and overdominant (*p* = 0.030) models. The odds of developing non-invasive PA are reduced by 2-fold under the dominant (*p* = 0.021) model for *TAS2R16* rs860170 CT + CC genotypes and by 2-fold under the additive (*p* = 0.018) model for each *TAS2R16* rs860170 C allele. The PA group had higher serum levels of TAS2R16 than the control group (*p* < 0.001). The present study found that patients with the *TAS2R16* rs978739 TT or CT genotype had higher serum TAS2R16 levels and protein concentrations than healthy individuals (*p* = 0.025 and *p* = 0.019, respectively), and those with the AA or AG genotype of *TAS2R16* rs1357949 had higher protein concentrations (*p* = 0.005 and *p* = 0.007, respectively). Conclusions: The *TAS2R16* rs978739 C allele was less common in the non-invasive PA group compared to the control group, while the *TAS2R16* rs860170 CT genotype was linked to a reduced likelihood of developing non-invasive PA. Additionally, the PA group showed higher serum levels of TAS2R16, and increased serum protein concentrations were observed in PA patients with specific *TAS2R16* variants.

## 1. Introduction

The pituitary gland is the main regulatory gland of the endocrine system, which transmits signals from the hypothalamus to target organs through hormone secretion [1]. The endocrine center is the adenohypophysis, whose specialized cells produce adrenocorticotropic (ACTH), follicle-stimulating (FSH), luteinizing (LH), thyroid-stimulating (TSH), and growth (GH) hormones and prolactin (PRL) [2]. The pituitary gland secretes hormones that control growth, reproduction, metabolism, and stress response, among other essential physiological processes [3]. Pituitary lesions, such as pituitary adenomas (PAs), may impair the activity of the pituitary gland [4]. PAs are usually benign tumors arising from the epithelial cells of the anterior pituitary gland. PA is one of the most common central nervous system tumors [5]. Research data show that adenomas’ population prevalence is  ~ 80/100,000 [4]. The etiology of PA is diverse; more than 95% of PA occurs sporadically, without any familial or hereditary cause [6]. Various inflammatory processes and immune responses can affect PAs’ occurrence [7,8]. The pathogenic mechanisms of pituitary tumors are complex and involve multiple factors, including interactions with environmental influences, genetic mutations, disrupted protein expression, and epigenetic changes [9]. Meanwhile, the accumulation of genetic mutations leads to oncogenic changes such as prolonged proliferation, invasion, angiogenesis, and resistance to cell death [10].

The *TAS2R16* (Taste 2 Receptor Member 16) gene, which codes for a taste receptor that is a member of the G protein-coupled receptor (GPCR) family, has been found to play a role in the inflammation process [11]. Specifically, studies have shown that activation of TAS2R (Taste 2 Receptor Member) can inhibit inflammatory responses, such as suppressing proinflammatory cytokine release [12]. This suggests that *TAS2R16* may have anti-inflammatory properties and could potentially be targeted for therapeutic purposes in conditions involving inflammation [11,12]. Mutations in the *TAS2R16* gene can result in TAS2R16 pro-activation that can trigger intracellular signaling pathways, such as the NF-kB pathway, which is crucial in regulating inflammatory responses and normal tissue homeostasis [11]. Since *TAS2R16* is involved in inflammatory processes, it could help modulate the chronic inflammation often associated with cancer progression [13]. TAS2R16 can influence the tumor microenvironment due to its expression in various immune cells, including macrophages and dendritic cells. In these cells, TAS2R16 can affect their activation state and function, thereby contributing to angiogenesis, tumor growth, and immune suppression [11,14,15]. Overall, chronic inflammation can contribute to the development of PAs by promoting abnormal cell growth and disrupting normal regulatory mechanisms within the pituitary gland [11,13,14,15]. However, inflammation is not the only cause, as genetic factors, hormonal imbalances, and other environmental influences also play significant roles in the formation of PA [9]. A recent study examined the association of *TAS2R16* single-nucleotide variants (SNVs) with colon cancer [16], as polymorphic variants of this gene have been shown to increase alcohol dependence [17]. Moreover, other research suggests that taste receptors, including *TAS2R16*, may also have roles in various non-taste-related biological processes, including cell proliferation and tumor growth [18]. It was also identified that activation of certain taste receptors, including TAS2R16, can induce apoptosis in cancer cells [14]. *TAS2R16* represents a promising target in PA development due to its potential roles in regulating cell proliferation, apoptosis, immune responses, and inflammation within the tumor microenvironment. Based on these associations of *TAS2R16,* we investigated the association of *TAS2R16* gene variants (rs860170, rs978739, rs1357949) and TAS2R16 serum levels with PA and its clinical manifestations.

## 2. Materials and Methods

This case–control study was conducted in accordance with the Declaration of Helsinki guidelines and received approval from the Kaunas Regional Biomedical Research Ethics Committee at the Lithuanian University of Health Sciences (Protocol No. BE-2-47). All participants were fully informed about this study and provided written informed consent. 

### 2.1. Selection of Subjects for This Study 

This study included patients with PA and a control group (healthy individuals). All subjects included in this study signed an informed consent form. The study participants were divided into PA patients and healthy individuals. For PA patients to be included in the study group, they had to meet the following criteria: the diagnosis of the disease was confirmed by magnetic resonance imaging (MRI), the age of the patients was ≥18 years, the patients did not have other brain cancers or tumors of different localization, there were no other undiagnosed diseases during the ophthalmological examination of the eye, and a signed informed consent form was provided. Meanwhile, subjects in the control group had to be healthy, and free from chronic and acute infectious and non-infectious diseases. Also, they had no history of pituitary adenoma or other tumors and were at least 18 years old. They had to sign an informed consent form to be included in this study. This study excluded individuals with opacities of the optical system, poor quality of fundus photography, and other systemic diseases (e.g., diabetes mellitus, cancer, systemic tissue disorders, and post-transplant conditions). 

All participants in this study were Lithuanians. The PA group consisted of 131 persons, of which 79 were women (60.3%) and 52 were men (39.7%). The age of the subjects was 19–80 years. The control group comprised 221 individuals, including 129 women (58.4%) and 92 men (41.6%), and their ages ranged from 18 to 88 years. The demographic data are shown in Table 1. 

### 2.2. SNV Selection

Our selection focused on single-nucleotide variants with known relevance to PA pathogenesis. SNVs may affect protein concentrations, leading to changes causing PA [19]. *TAS2R16* mutations have the potential to increase protein levels, which, in turn, can activate intracellular signaling pathways that are essential for controlling inflammatory responses and maintaining normal tissue homeostasis [11]. According to recent studies, *TAS2R16* may potentially be involved in a number of biological processes unrelated to taste, such as tumor formation and cell proliferation [18]. Some research suggests that activating *TAS2R16* and other taste receptors can induce apoptosis in cancer cells [14]. Because *TAS2R16* may have a role in immune response, tumor microenvironment inflammation, cell proliferation, apoptosis, and other processes, it is a prospective target in the development of PA. The gene selection for our study was informed by prior research, highlighting their associations with other cancers and inflammatory processes [12,14,16]. *TAS2R16* was chosen because its variants affect the infection process. Specifically, rs860170, rs978739, and rs1357949 variants in the *TAS2R16* gene were selected for this study. The *TAS2R16* rs978739 variant is a mutation in the promoter region of the gene in which thymine is replaced by cytosine (T > C) [20], while rs860170 is a missense variant in which cytosine is replaced by thymine (C > T) [21]. rs1357949 is a mutation in which adenine is changed to guanine (A > G) [22]. 

### 2.3. DNA Isolation

Peripheral blood, collected in vacuum tubes containing EDTA (ethylenediaminetetraacetate), was used for DNA isolation. Using these tubes, the DNA is protected from degradation and clot formation. From white blood cells, the genomic DNA was isolated using the salt precipitation method. It is crucial to note that all DNA extraction steps should be performed at low temperatures (approximately 4 °C). It is recommended to store the isolated DNA at −20 °C before use. This salt precipitation method involves collecting cells by centrifugation, retaining them in a buffer solution, disrupting cell membranes with detergents, hydrolyzing proteins, removing protein with chloroform, and precipitating DNA with ethanol.

### 2.4. Genotyping

In order to genotype *TASR216* (rs860170, rs978739, and rs1357949), the real-time polymerase chain reaction (RT-PCR) assay was employed. Genotypes of rs860170, rs978739, and rs1357949 SNVs in 352 test samples were determined by using the RT-PCR amplifier StepOne Plus (Applied Biosystems, Singapore). The primers and molecular markers used for genotyping were also from the company Applied Biosystem by Thermofisher Scientifics (Thermo Scientific, Waltham, MA, USA). A total of 1.5 µL (10 ng) of genomic DNA from subjects and 8.5 µL of the PCR reaction mixture were used for each reaction. The composition of the PCR mixture was prepared using TaqMan Universal Master Mix II (Applied Biosystems by Thermofisher Scientific, Vilnius, Lithuania), nuclease-free water (Invitrogen by Thermo Fisher Scientific, Paisley, JK), and genotyping kits for all three SNVs: rs860170 (C___8560176_40), rs978739 (C___2618720_10), and rs1357949 (C___8560175_10) (Applied Biosystems by Thermofisher Scientifics, Pleasanton, CA, JAV). Genotyping was performed according to the manufacturer’s recommendations.

### 2.5. TAS2R16 Protein Concentration Measurement

Peripheral venous blood was drawn and incubated at room temperature for 30 min before centrifugation to prepare the serum. After centrifugation, the serum was extracted from the pellet, put into 2 mL containers, and refrigerated at −80 °C until analysis. The protein concentrations of TAS2R16 in blood serum were tested in duplicate in 20 PA patients and 20 control subjects. The enzyme-linked immunosorbent assay (ELISA) was performed for this study, and the Abbexa Human Taste Receptor Type 2 Member 16 (TAS2R16) ELISA kit (Abbexa LTD; Cambridge, UK) was used. The standard curve for TAS2R16 protein levels in the serum has a sensitivity of less than 0.1 ng/mL and a range of 0.312–20 ng/mL. The test was carried out following the manufacturer’s instructions. The optical density at 450 nm was measured using a Multiscan FC Microplate Photometer (Thermo Scientific, Waltham, MA, USA). The protein concentration of TAS2R16 was determined using a standard curve. 

### 2.6. Statistical Analysis

According to the power calculator [23], using the minor allele frequencies of all of the selected SNVs, which were ≥10%, and the global PA prevalence (17%) [24], we determined that the sample size should be at least about 100 subjects in a group. We confirmed that our collected sample sizes for the PA and control groups were sufficient to reach 80% or higher power for the selected SNV analysis. 

IBM SPSS Statistics 29.0 was used for the analysis of statistical data. The data are shown as the median (interquartile range (IQR)) and actual numbers (percentages). Utilizing the non-parametric Mann–Whitney U test, the age differences between the two independent groups were ascertained. When the data were not normally separated, Pearson’s χ² test was used to compare the homogeneity of the distribution of *TAS2R16* variants between patients with PA and the control group. Binomial logistic regression analysis estimated the odds ratio (OR) for the occurrence of PA, considering the genotypes according to the constructed inheritance models. Binomial logistic regression analysis was also performed for individual PA groups considering PA activity, invasiveness, and recurrence, indicating the OR with a 95% confidence interval (CI). The logistic regression analysis was performed using various genetic models: codominant (heterozygotes versus wild-type homozygotes and minor allele homozygotes versus wild-type homozygotes), dominant (minor allele homozygotes and heterozygotes versus wild-type homozygotes), recessive (minor allele homozygotes versus wild-type homozygotes and heterozygotes), and overdominant (heterozygotes versus wild-type homozygotes and minor allele homozygotes). Additionally, the additive model was employed to assess the impact of each minor allele on PA.

Haplotype analysis of *TAS2R16* (rs860170, rs978739, rs1357949) was also performed using the online calculator “SNPStats” [25], and the influence of D’, r^2^, and haplotypes on the occurrence of PA was evaluated. Differences were considered statistically significant when *p* < 0.05. We also applied a Bonferroni correction (0.05/3, since we assessed three SNPs in the *TAS2R16* gene). 

## 3. Results

### 3.1. Associations of TAS2R16 rs860170, rs978739, and rs1357949 with Pituitary Adenoma

We found no statistically significant differences between the PA and control groups’ genotype and allele frequency distributions (Supplementary Materials, Table S1). Additionally, no statistically significant findings were obtained from the binomial logistic regression analysis (Supplementary Materials, Table S2).

### 3.2. Associations of TAS2R16 rs860170, rs978739, and rs1357949 with Pituitary Adenoma in Women and Men Groups

Male and female analyses were conducted independently; however, neither the genotype/allele distribution nor the binomial logistic regression showed statistically significant findings (Supplementary Materials, Tables S3–S5).

### 3.3. Associations of TAS2R16 rs860170, rs978739, and rs1357949 with Pituitary Adenoma’s Recurrence

There were no statistically significant differences in genotype and allele distributions between the PA group with recurrence and the control group, or between the PA group without recurrence and the control group (Supplementary Materials, Table S6). Additionally, the binomial logistic regression analysis did not yield statistically significant results (Supplementary Materials, Tables S7 and S8). 

### 3.4. Associations of TAS2R16 rs860170, rs978739, and rs1357949 with Pituitary Adenoma’s Activeness

The differences between both the non-active PA group and the control group, and between the active PA group and the control group were analyzed for their adenomas’ activeness distribution of genotypes and alleles. Nevertheless, no statistically significant results were found by the binomial logistic regression analysis or the distribution analysis (Supplementary Materials, Tables S9–S11).

### 3.5. Associations of TAS2R16 rs860170, rs978739, and rs1357949 with Pituitary Adenoma’s Invasiveness

The differences between both the non-invasive PA group and the control group, and between the invasive PA group and the control group were analyzed for their adenomas’ invasiveness distribution of genotypes and alleles. The results showed that the *TAS2R16* rs978739 C allele is less frequent in the non-invasive PA group compared to the control group (24.2 % vs. 32.8 %, *p* = 0.045), but this result did not reach the Bonferroni-corrected significance level (Table 2).

The binomial logistic regression analysis determined the impact of *TAS2R16* rs860170, rs978739, and rs1357949 on non-invasive and invasive PA development. The analysis revealed that for *TAS2R16* rs860170 under the codominant and overdominant model, the CT genotype reduces the odds of developing non-invasive PA by 1.9-fold (OR: 0.519, CI: 0.293–0.917, *p* = 0.024 and OR: 0.532, CI: 0.301-0.939, *p* = 0.030, respectively). However, *TAS2R16* rs860170 CT+CC genotypes decrease the odds of developing non-invasive PA by 2-fold under the dominant model (OR: 0.511, CI: 0.289-0.904, *p* = 0.021), and each of *TAS2R16* rs860170 C allele decreases these odds by 2-fold under the additive model (OR: 0.506, CI: 0.289-0.888, *p* = 0.018) (Table 3). Also, none of these results survived Bonferroni correction. The binomial logistic regression analysis performed on the invasive PA and control groups did not reveal statistically significant results (Supplementary Materials, Table S12).

### 3.6. Haplotype Analysis of TAS2R16 rs860170, rs978739, and rs1357949

The square of the haplotype frequency correlation coefficient (r^2^) was 0.167, and the difference (D’) between the predicted and actual haplotype frequencies was computed to be 0.962. The most prevalent haplotype in the research group, TAS2R16 rs860170-T-rs978739-T-rs1357949-A, was chosen as the reference. The haplotype analysis did not show statistically significant associations with the occurrence of PA (Supplementary Materials, Table S13). 

### 3.7. TAS2R16 Serum Levels’ Association with PA

**Figure 1:**
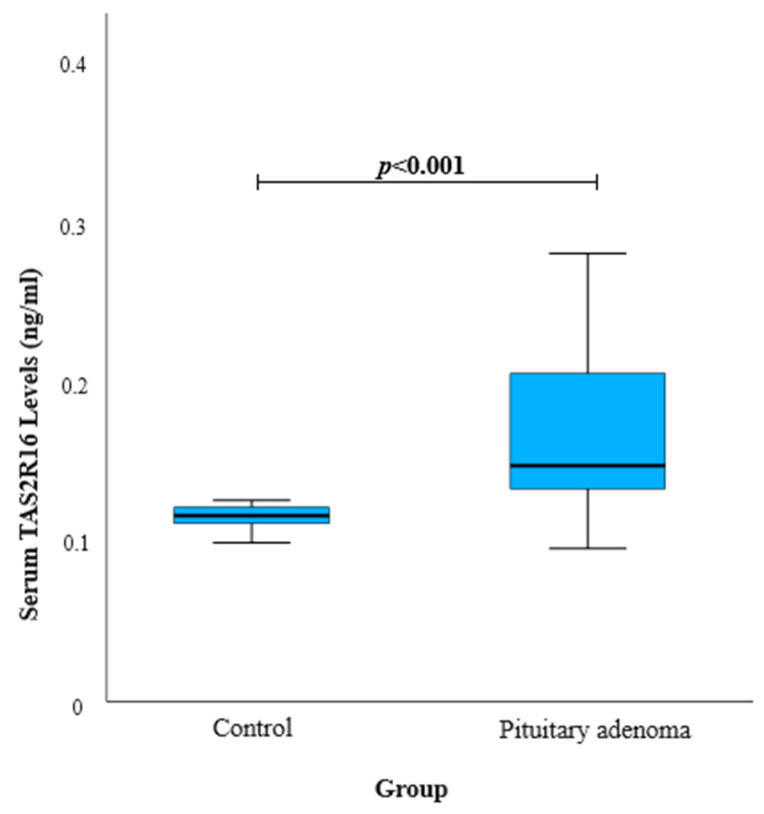
TAS2R16 serum levels in PA and control groups.

Serum levels of TAS2R16 were measured twice for 20 PA patients and 20 control participants. TAS2R16 serum levels were found to be higher in the PA group than in the control group, with median values (IQR) of 0.116 (0.012) and 0.147 (0.075) ng/mL, respectively (*p* < 0.001) (Figure 1).

Furthermore, a comparison of TAS2R16 concentration was carried out across various genotypes of the *TAS2R16* SNVs rs860170, rs978739, and rs1357949 (Table 4). Serum protein levels were greater in PA patients with the TT or CT genotype of the *TAS2R16* rs860170 SNV than in the healthy control group (*p* = 0.031 and *p* = 0.006, respectively). *TAS2R16* rs978739 SNV analysis revealed that blood serum levels of TAS2R16 were higher in PA patients with the TT or CT genotype than in healthy participants (*p* = 0.025 and 0.019, respectively). Additionally, TAS2R16 protein concentrations were greater in PA patients with the AA or AG genotype of the *TAS2R16* rs1357949 SNV than in healthy persons (*p* = 0.005 and *p* = 0.007, respectively). 

## 4. Discussion

We investigated *TAS2R16* gene variants rs860170, rs978739, and rs1357949 in 131 PA patients and 221 control subjects. These variants have not been investigated in studies analyzing PA development, activity, invasiveness, and recurrence.

Although we found no association between *TAS2R16* rs860170, rs978739, and rs1357949 and PA development, further analysis showed that the *TAS2R16* rs978739 C allele is statistically rarer in the non-invasive PA group than in the control group (24.2 % vs. 32.8 %, *p* = 0.045). As mentioned before, no studies have been related to the association between rs978739 and PA. However, there has been a study looking for associations with colon cancer. Barontini and co-authors did not find a statistically significant difference with this type of cancer when studying the rs978739 A/G variant (*p* = 0.65) [16]. rs978739 located in the *TAS2R16* promoter region was investigated, suggesting that this SNV is important for regulating the protein and plays a role in the longevity process [18,26]. Camba and co-authors studied 941 individuals from Calabria, Italy, whose ages ranged from 20 to 106 years. Scientists found that the rs978739 variant showed a statistically significant association with longevity (*p* = 0.001). Specifically, the proportion of individuals with the homozygous AA genotype increased over time, rising from 35% in people between the ages of 20 and 70 to 55% in people over the age of 100 [26]. Another group of researchers decided to conduct a meta-analysis to reconcile previous studies with inconsistent results and determine whether rs978739 is associated with longevity. The rs978739 TT genotype is significantly associated with longevity in the Calabrian population, maintaining its significance in a meta-analysis with data from the Cilento population, which was insignificant in this study. Therefore, the obtained results strengthen the assumption that the *TAS2R16* rs978739 TT genotype is associated with longevity in Southern Italy [18]. Meanwhile, non-invasive PAs are simpler to treat and maintain since they are usually isolated and have not spread to nearby tissues. Patients with non-invasive cancer may have an increased life expectancy and have better prognoses with early discovery and effective treatment [5,27]. 

Analysis of another SNV, *TAS2R16* rs1357949, revealed no statistically significant difference between the PA subgroups (divided by recurrence, activity, or invasiveness). Other studies have examined the influence of this *TAS2R16* rs1357949 variant on glucose homeostasis. Because of the importance of taste receptors in regulating food intake and food response to chemo stimuli, it has been suggested that differences in the efficacy of taste receptors may influence the maintenance of steady blood glucose levels. No statistically significant difference in glucose homeostasis was found when studying rs1357949 (*p* = 0.50) [28]. Also, the association of *TAS2R16* rs1357949 with colon cancer was investigated, but the researchers failed to detect statistically significant associations (*p* = 0.31) [16]. The previously mentioned study by Camba and co-authors also examined the effect of rs1357949 on longevity. Although this SNV was not statistically significantly associated with longevity, the *TAS2R16* gene haplotype (rs1357949–rs6466849–rs860170–rs978739: T_A_A_G) showed an association with longevity (OR: 0.74, 95% CI: 0.56–0.98, *p* = 0.03) [26]. Scientists also investigated the link between multiple sclerosis (MS) and *TAS216* SNVs [29]. Pituitary adenoma and MS have been identified in many patients. Numerous studies have been interested in attempting to determine the association between the two central nervous system conditions, although there is still debate regarding a clear causal relationship [30]. Scientists discovered that the rs860170, rs978739, and rs1357949 C-C-A haplotype was linked to a ~12-fold greater chance of developing MS (*p* = 0.02) [29]. Our study also examined haplotypes composed of rs860170, rs978739, and rs1357949 SNVs that would predispose individuals to the occurrence of PA. Unfortunately, the haplotype analysis did not reveal any statistically significant associations with PA.

However, the binomial logistic regression analysis indicated that the TAS2R16 rs860170 CT genotype reduced the odds of developing non-invasive PA by 1.9-fold under both the codominant (*p* = 0.024) and overdominant (*p* = 0.030) genetic models. Also, *TAS2R16* rs860170 CT+CC genotypes lower the chances of developing non-invasive PA by 2-fold under the dominant model (*p* = 0.021), and each of *TAS2R16* rs860170 C alleles decreases these odds by 2-fold under the additive model (*p* = 0.018). Other researchers investigated the relationship between *TAS2R16* rs860170 and colon and rectal cancers. However, they could not find any statistically significant correlations (*p* = 0.37) [16]. The relationship between MS and *TAS2R16* rs860170 was also analyzed. According to the analyses between the MS and control groups, there was a statistically significant increase (*p* < 0.001) in the frequency of the *TAS2R16* rs860170 CC genotype and a statistically significant difference (*p* = 0.041) in the frequency of the *TAS2R16* rs860170 TT genotype. According to the findings, in the codominant model, the CC genotype increased the odds of MS by around 27 times (*p* = 0.002), and for each C allele, the odds increased by 1.8 times (*p* < 0.001) [29]. Currently, there is limited research on *TAS2R16* in CNS diseases and brain tumors, particularly in PA. Further studies with larger sample sizes are needed to better understand the gene’s role in the initiation, growth, and activity of pituitary tumors. Although our study did not find associations between *TAS2R16* gene variants (rs860170, rs978739, and rs1357949) and PA, there may be other *TAS2R16* gene genetic variations that may be associated with PA generally. For example, another *TAS2R16* variant (rs1525489) was investigated in a study by Barontini and co-authors, whose C allele was associated with an increased risk of rectal cancer (*p* = 0.047) [16]. One of the risk factors for both colon cancer and PA is known to be heavy alcohol consumption [17,31], which is influenced by *TAS2R16* genetic variations associated with alcohol dependence [32]. 

TAS2R16 has been found in various tissues beyond the tongue, including in the brain and gastrointestinal tract [33]. We observed that TAS2R16 serum levels were significantly higher in the PA group than in the control group (*p* < 0.001). Additionally, serum levels were elevated in PA patients with either the TT or CT genotype of *TAS2R16* rs860170 than in the control group (*p* = 0.031 and *p* = 0.006, respectively). Our analysis revealed that blood serum levels of TAS2R16 were higher in PA patients with the *TAS2R16* rs978739 CT or TT genotype than in control group individuals (*p* = 0.019 and *p* = 0.025, respectively). Additionally, TAS2R16 protein concentrations were greater in PA patients with the AA or AG genotype (*p* = 0.005 and *p* = 0.007, respectively) of *TAS2R16* rs1357949 compared to the healthy subjects. These findings suggest that elevated TAS2R16 concentrations could alter the dynamics of local cellular processes in PA patients. Also, studies indicate that TAS2R16 might play a role in modulating immune responses [13,18]. TAS2R16 could contribute to the inflammatory environment that sometimes accompanies PA, potentially affecting tumor growth or the body’s response to the tumor. Given that TAS2R16 could be involved in specific cellular signaling pathways that influence cell proliferation, apoptosis, or hormone secretion within pituitary cells [14,18], any disruption of these pathways, such as increased TAS2R16 concentration in serum, could contribute to the development or progression of PA. However, further research is needed to fully understand the clinical relevance of elevated serum levels of TAS2R16 in PA.

TAS2R16 serum levels were noted by several researchers in MS patients. Their analysis revealed that the MS group’s serum TAS2R16 levels were higher than those of the healthy participants (*p* = 0.014) [29]. 

## 5. Conclusions

We can hypothesize that *TAS2R16* genetic variants may influence the occurrence of PA, as they are associated with the development of rectal cancer. Further studies investigating the association between *TAS2R16* rs860170, rs978739, and rs1357949 and PA and other risk factors should be performed to confirm this hypothesis. Also, our study found that the *TAS2R16* rs978739 C allele is statistically rarer in non-invasive PA patients than in healthy individuals. Also, our results showed that each C allele of the rs978739 variant lowers the chance of developing a non-invasive form of PA. This can suggest that the rs978739 C allele plays a protective role in the invasiveness of PA. Moreover, the PA patients’ serum TAS2R16 levels were elevated compared to the control group. This could indicate that TAS2R16 as a molecule plays a role in the pathogenesis of PA. On the other hand, one key limitation of our study is the small sample size within certain subgroups, which reduces the statistical power and increases the risk of Type II errors. Specifically, the small number of participants in our protein concentration analysis (20 PA patients and 20 controls) limits the generalizability of our findings. This may also affect the reliability of the subgroup analyses, such as those based on gender or disease characteristics. Moreover, environmental exposures, lifestyle factors, or other genetic variations should be included in future studies with larger sample sizes to improve the results.

## Figures and Tables

**Table 1:** The demographic data of the participants of this study.

Characteristics	PA Group (n = 131)	Control Group (n = 221)	*p*-Value
Women, n (%)	79 (60.3)	129 (58.4)	0.721 *
Men, n (%)	52 (39.7)	92 (41.6)	
Age, median (IQR)	54 (21)	53 (23)	0.592 **
Recurrence: With recurrence, n (%) Without recurrence, n (%)	33 (25.2) 98 (74.8)		
Invasiveness: Not invasive, n (%) Invasive, n (%)	62 (47.3) 69 (52.7)		
Hormone activity: Not active, n (%) Active, n (%)	56 (42.7) 75 (57.3)		

* Chi-Square; ** Mann–Whitney U test; *p*-value: significance level (statistically significant when *p* < 0.017).

**Table 2:** Distributions of *TAS2R16* rs860170, rs978739, and rs1357949 genotypes, and alleles in patients with PA and those in control group by PA invasiveness.

Genotype/ Allele	Control Group, n (%) (n = 221)	Invasive PA Group, n (%) (n = 69)	*p*-Value	Non-Invasive PA Group, n (%) (n = 62)	*p*-Value
*TAS2R16* rs860170					
TT	78 (35.3)	26 (37.7)	0.696	32 (51.6)	0.056
CT	141 (63.8)	43 (62.3)		30 (48.4)	
CC	2 (0.9)	0 (0)		0 (0)	
T	297 (67.2)	95 (68.8)	0.718	94 (75.8)	0.066
C	145 (32.8)	43 (31.2)		30 (24.2)	
*TAS2R16* rs978739					
TT	123 (55.7)	38 (55.1)	0.203	27 (43.5)	0.137
CT	83 (37.6)	30 (43.5)		27 (43.5)	
CC	15 (6.8)	1 (1.4)		8 (12.9)	
T	329 (74.4)	106 (76.8)	0.573	81 (65.3)	0.045
C	113 (25.6)	32 (23.2)		43 (34.7)	
*TAS2R16* rs1357949					
AA	92 (41.6)	31 (44.9)	0.649	24 (38.7)	0.828
AG	106 (48.0)	29 (42.0)		30 (48.4)	
GG	23 (10.4)	9 (13.0)		8 (12.9)	
A	290 (65.6)	91 (65.9)	0.943	78 (62.9)	0.576
G	152 (34.4)	47 (34.1)		46 (37.1)	

*p*-value: significance level (statistically significant when *p* < 0.017).

**Table 3:** Binomial logistic regression analysis of *TAS2R16* rs860170, rs978739, and rs1357949 in non-invasive PA group and control group.

Model	Genotype/Allele	OR (95% CI)	*p*-Value	AIC
*TAS2R16* rs860170				
Additive	C	0.506 (0.289–0.888)	0.018	293.890
Codominant	CT vs. TT CC vs. TT	0.519 (0.293–0.917)	0.024	295.479
Dominant	CT + CC vs. TT	0.511 (0.289–0.904)	0.021	294.245
Overdominant	CT vs. TT + CC	0.532 (0.301–0.939)	0.030	294.839
Recessive	CC vs. CT + TT			
*TAS2R16* rs978739				
Additive	C	1.532 (1.001–2.345)	0.050	295.765
Codominant	CT vs. TT CC vs. TT	1.482 (0.812–2.705) 2.430 (0.936–6.306)	0.200 0.068	297.741
Dominant	CT + CC vs. TT	1.627 (0.922–2.871)	0.093	296.723
Overdominant	CT vs. TT + CC	1.283 (0.725–2.270)	0.393	298.845
Recessive	CC vs. CT + TT	2.035 (0.820–5.049)	0.126	297.379
*TAS2R16* rs1357949				
Additive	G	1.133 (0.740–1.737)	0.565	299.241
Codominant	AG vs. AA GG vs. AA	1.085 (0.592–1.987) 1.333 (0.531–3.350)	0.792 0.541	301.202
Dominant	AG + GG vs. AA	1.129 (0.634–2.010)	0.680	299.399
Overdominant	AG vs. AA + GG	1.017 (0.579–1.787)	0.953	299.567
Recessive	GG vs. AA + AG	1.275 (0.540–3.011)	0.579	299.272

OR: odds ratio; CI: confidence interval; AIC: Akaike information criterion; *p*-value: significance level (statistically significant when *p* < 0.017).

**Table 4:** Distribution of *TAS2R16* rs860170, rs978739, and rs1357949 genotypes in PA and control groups according to TAS2R16 serum concentration.

Genotype	PA Group	Control Group	*p*-Value
*TAS2R16* rs860170			
TT	0.183 ^1^ (0.074) ^2^	0.118 ^1^ (0.012) ^2^	0.031 *
CT	0.144 ^3^ (0.066) ^4^	0.117 ^3^ (0.013) ^4^	0.006 **
CC	NA	NA	NA
*TAS2R16* rs978739			
TT	0.144 ^3^ (0.096) ^4^	0.117 ^3^ (0.0166) ^4^	0.025 **
CT	0.150 ^3^ (0.077) ^4^	0.110 ^3^ (0.0150) ^4^	0.019 **
CC	NA	NA	NA
*TAS2R16* rs1357949			
AA	0.159 ^3^ (0.087) ^4^	0.118 ^3^ (0.014) ^4^	0.005 **
AG	0.138 ^3^ (0.027) ^4^	0.115 ^3^ (0.010) ^4^	0.007 **
GG	NA	NA	NA

^1^ mean; ^2^ standard deviation; ^3^ median; ^4^ interquartile range (IQR); * Student’s *t* test; ** Mann–Whitney U test; *p*-value: significance level (statistically significant when *p* < 0.05); NA—not applicable.

## Data Availability

The data can be shared up on request.

## Supplementary Materials

The following are available online at https://www.mdpi.com/article/10.3390/biomedicines12092022/s1: Table S1: Distributions of *TAS2R16* rs860170, rs978739, rs1357949 genotypes and alleles in patients with PA and control group; Table S2: Binomial logistic regression of *TAS2R16* rs860170, rs978739, rs1357949 in patients with PA and control group; Table S3: Distributions of *TAS2R16* rs860170, rs978739, rs1357949 genotypes, and alleles in patients with PA and control group by gender; Table S4: Binomial logistic regression of *TAS2R16* rs860170, rs978739, rs1357949 in women with PA and women of the control group; Table S5: Binomial logistic regression of *TAS2R16* rs860170, rs978739, rs1357949 in men with PA and men of the control group; Table S6: Distributions of *TAS2R16* rs860170, rs978739, rs1357949 genotypes, and alleles in patients with PA and control group by PA recurrence; Table S7: Binomial logistic regression analysis of *TAS2R16* rs860170, rs978739, and rs1357949 in the PA without recurrence group and control group; Table S8: Binomial logistic regression analysis of *TAS2R16* rs860170, rs978739, and rs1357949 in the PA with recurrence group and control group; Table S9: Distributions of *TAS2R16* rs860170, rs978739, rs1357949 genotypes, and alleles in patients with PA and control group by PA activeness; Table S10: Binomial logistic regression analysis of *TAS2R16* rs860170, rs978739, and rs1357949 in the not active PA group and control group; Table S11: Binomial logistic regression analysis of *TAS2R16* rs860170, rs978739, and rs1357949 in the active PA group and control group; Table S12: Binomial logistic regression analysis of *TAS2R16* rs860170, rs978739, and rs1357949 in the invasive PA group and control group; Table S13: Haplotype association of *TAS2R16* rs860170, rs978739, rs1357949 with the predisposition to PA development.
