# Supplementary material for: Exploring the Role of the TAS2R16 Protein and Its Single Nucleotide Variants in Pituitary Adenoma Development
Source: Biomedicines. 2024 Sep 4;12(9):2022. doi: 10.3390/biomedicines12092022 (PMC11429365; doi:10.3390/biomedicines12092022)
Supplement: Supplementary file 1 [file biomedicines-12-02022-s001.zip › biomedicines-3149474-supplementary.pdf]

Table S1. Distributions of TAS2R16 rs860170, rs978739, rs1357949 genotypes and alleles in patients with PA and control group

**Genotype/allele**	**PA group, n (%) (n=131)**	**Control group, n (%) (n=221)**	***p*-value**
TAS2R16 rs860170			
TT	58 (44.3)	78 (35.3)	0.151
CT	73 (55.7)	141 (63.8)	
CC	0 (0)	2 (0.9)	
T	189 (72.1)	297 (67.2)	0.170
C	73 (27.9)	145 (32.8)	
TAS2R16 rs978739			
TT	65 (49.6)	123 (55.7)	0.523
CT	57 (43.5)	83 (37.6)	
CC	9 (6.9)	15 (6.8)	
T	187 (71.4)	329 (74.4)	0.375
C	75 (28.6)	113 (25.6)	
TAS2R16 rs1357949			
AA	55 (42.0)	92 (41.6)	0.730
AG	59 (45.0)	106 (48.0)	
GG	17 (13.0)	23 (10.4)	
A	169 (64.5)	290 (65.6)	0.766
G	93 (35.5)	152 (34.4)	

p-value: significance level (statistically significant when $p < 0.017$)

Table S2. Binomial logistic regression of TAS2R16 rs860170, rs978739, rs1357949 in patients with PA and control group

**Model**	**Genotype/allele**	**OR (95 % CI)**	***p*-value**	**AIC**
TAS2R16 rs860170				
Additive	C	0.671 (0.435–1.036)	0.072	463.465
Codominant	CT *vs.*TT<br>CC *vs.*TT	0.696 (0.448–1.083)	0.108	464.265
Dominant	CT+CC *vs.*TT	0.687 (0.441–1.068)	0.095	461.924
Overdominant	CT *vs.* TT+CC	0.714 (0.460–1.109)	0.134	464.467
Recessive	CC *vs.* CT+TT			
TAS2R16 rs978739				
Additive	C	1.171 (0.828–1.655)	0.372	465.911
Codominant	CT *vs.*TT<br>CC *vs.*TT	1.300 (0.827–2.041)<br>1.135 (0.471–2.736)	0.256<br>0.777	467.414
Dominant	CT+CC *vs.*TT	1.274 (0.826–1.966)	0.273	465.503
Overdominant	CT *vs.* TT+CC	1.281 (0.825–1.988)	0.270	465.493
Recessive	CC *vs.* CT+TT	1.013 (0.430–2.385)	0.976	466.706
TAS2R16 rs1357949				

Additive	G	1.052 (0.759–1.457)	0.762	466.615
Codominant	AG *vs.* AA<br>GG *vs.* AA	0.931 (0.587–1.477)<br>1.236 (0.608–2.516)	0.762<br>0.558	468.083
Dominant	AG+GG *vs.* AA	0.985 (0.636–1.527)	0.948	466.702
Overdominant	AG *vs.* AA+GG	0.889 (0.576–1.372)	0.595	466.424
Recessive	GG *vs.* AA+AG	1.284 (0.658–2.503)	0.464	466.175

OR: odds ratio; CI: confidence interval; AIC: Akaike information criterion; *p*-value: significance level (statistically significant when $p < 0.017$).

Table S3. Distributions of TAS2R16 rs860170, rs978739, rs1357949 genotypes, and alleles in patients with PA and control group by gender

**Genotype/ allele**	**Women**		***p*-value**	**Men**		***p*-value**
	**PA group, n (%) (n=79)**	**Control group, n (%) (n=129)**		**PA group, n (%) (n=52)**	**Control group, n (%) (n=92)**	
TAS2R16 rs860170						
TT	35 (44.3)	51 (39.5)	0.602	23 (44.2)	27 (29.3)	0.160
CT	44 (55.7)	77 (59.7)		29 (55.8)	64 (69.6)	
CC	0 (0)	1 (0.8)		0 (0)	1 (1.1)	
T	114 (72.2)	179 (69.4)	0.548	75 (72.1)	118 (64.1)	0.166
C	44 (27.8)	79 (30.6)		29 (27.9)	66 (35.9)	
TAS2R16 rs978739						
TT	39 (49.4)	70 (54.3)	0.655	26 (50.0)	53 (57.6)	0.649
CT	35 (44.3)	49 (38.0)		22 (42.3)	34 (37.0)	
CC	5 (6.3)	10 (7.8)		4 (7.7)	5 (5.4)	
T	113 (71.5)	189 (73.3)	0.699	74 (71.2)	140 (76.1)	0.357
C	45 (28.5)	69 (26.7)		30 (28.8)	44 (23.9)	
TAS2R16 rs1357949						
AA	32 (40.5)	56 (43.4)	0.915	23 (44.2)	36 (39.1)	0.449
AG	37 (46.8)	58 (45.0)		22 (42.3)	48 (52.2)	
GG	10 (12.7)	15 (11.6)		7 (13.5)	8 (8.7)	
A	101 (63.9)	170 (65,9)	0.891	68 (65.4)	120 (65.2)	0.977
G	57 (36.1)	88 (34.1)		36 (34.6)	64 (34.8)	

p-value: significance level (statistically significant when $p < 0.017$).

Table S4. Binomial logistic regression of TAS2R16 rs860170, rs978739, rs1357949 in women with PA and women of the control group

**Model**	**Genotype/allele**	**OR (95 % CI)**	***p*-value**	**AIC**
TAS2R16 rs860170				
Additive	C	0.802 (0.459–1.402)	0.439	277.613
Codominant	CT *vs.*TT	0.833 (0.472–1.469)	0.527	278.854

	CC *vs.*TT			
Dominant	CT+CC *vs.*TT	0.822 (0.466–1.449)	0.498	277.753
Overdominant	CT *vs.* TT+CC	0.849 (0.482–1.496)	0.571	277.891
Recessive	CC *vs.* CT+TT			
TAS2R16 rs978739				
Additive	C	1.092 (0.700–1.706)	0.698	278.061
Codominant	CT *vs.*TT<br>CC *vs.*TT	1.282 (0.715–2.300)<br>0.897 (0.286–2.814)	0.405<br>0.853	279.367
Dominant	CT+CC *vs.*TT	1.217 (0.695–2.132)	0.493	277.741
Overdominant	CT *vs.* TT+CC	1.299 (0.735–2.294)	0.368	277.401
Recessive	CC *vs.* CT+TT	0.804 (0.264–2.445)	0.701	278.061
TAS2R16 rs1357949				
Additive	G	1.091 (0.720–1.653)	0.682	278.044
Codominant	AG *vs.* AA<br>GG *vs.* AA	1.116 (0.613–2.032)<br>1.167 (0.469–2.900)	0.719<br>0.740	280.033
Dominant	AG+GG *vs.* AA	1.127 (0.638–1.989)	0.681	278.042
Overdominant	AG *vs.* AA+GG	1.078 (0.615–1.891)	0.792	276.142
Recessive	GG *vs.* AA+AG	1.101 (0.469–2.588)	0.825	278.163

OR: odds ratio; CI: confidence interval; AIC: Akaike information criterion; *p*-value: significance level (statistically significant when $p < 0.017$).

Table S5. Binomial logistic regression of TAS2R16 rs860170, rs978739, rs1357949 in men with PA and men of the control group

**Model**	**Genotype/allele**	**OR (95 % CI)**	***p*-value**	**AIC**
TAS2R16 rs860170				
Additive	C	0.514 (0.256–1.032)	0.061	186.839
Codominant	CT *vs.*TT<br>CC *vs.*TT	0.532 (0.262–1.080)	0.081	188.418
Dominant	CT+CC *vs.*TT	0.524 (0.258–1.063)	0.073	187.160
Overdominant	CT *vs.* TT+CC	0.552 (0.273–1.116)	0.098	187.633
Recessive	CC *vs.* CT+TT			
TAS2R16 rs978739				
Additive	C	1.297 (0.748–2.250)	0.354	189.512
Codominant	CT *vs.*TT<br>CC *vs.*TT	1.319 (0.647–2.690)<br>1.631 (0.404–6.587)	0.446<br>0.492	191.507
Dominant	CT+CC *vs.*TT	1.359 (0.686–2.690)	0.379	189.592
Overdominant	CT *vs.* TT+CC	1.251 (0.625–2.505)	0.527	189.969
Recessive	CC *vs.* CT+TT	1.450 (0.372–5.656)	0.593	190.086
TAS2R16 rs1357949				
Additive	G	0.992 (0.587–1.676)	0.976	190.367
Codominant	AG *vs.* AA<br>GG *vs.* AA	0.717 (0.347–1.484)<br>1.370 (0.437–4.288)	0.370<br>0.589	190.779
Dominant	AG+GG *vs.* AA	0.811 (0.407–1.614)	0.550	190.011
Overdominant	AG *vs.* AA+GG	0.672 (0.339–1.334)	0.256	189.069

Recessive	GG *vs.* AA+AG	1.633 (0.556–4.796)	0.372	189.582

OR: odds ratio; CI: confidence interval; AIC: Akaike information criterion; *p*-value: significance level (statistically significant when $p < 0.017$).

Table S6. Distributions of TAS2R16 rs860170, rs978739, rs1357949 genotypes, and alleles in patients with PA and control group by PA recurrence

**Genotype/ allele**	**Control group, n (%) (n=221)**	**PA group with recurrence, n (%) (n=33)**	***p*-value**	**PA group without recurrence, n (%) (n=98)**	***p*-value**
TAS2R16 rs860170					
TT	78 (35.3)	17 (51.5)	0.182	41 (41.8)	0.365
CT	141 (63.8)	16 (48.5)		57 (58.2)	
CC	2 (0.9)	0 (0)		0 (0)	
T	297 (67.2)	50 (75.8)	0.163	139 (70.9)	0.351
C	145 (32.8)	16 (24.2)		57 (29.1)	
TAS2R16 rs978739					
TT	123 (55.7)	19 (57.6)	0.830	46 (46.9)	0.287
CT	83 (37.6)	11 (33.3)		46 (46.9)	
CC	15 (6.8)	3 (9.1)		6 (6.1)	
T	329 (74.4)	49 (74.2)	0.973	138 (70.4)	0.289
C	113 (25.6)	17 (25.8)		58 (29.6)	
TAS2R16 rs1357949					
AA	92 (41.6)	13 (39.4)	0.719	42 (42.9)	0.831
AG	106 (48.0)	15 (45.5)		44 (44.9)	
GG	23 (10.4)	5 (15.2)		12 (12.2)	
A	290 (65.6)	41 (62.1)	0.579	128 (65.3)	0.940
G	152 (34.4)	25 (37.9)		68 (34.7)	

p-value: significance level (statistically significant when $p < 0.017$).

Table S7. Binomial logistic regression analysis of TAS2R16 rs860170, rs978739, and rs1357949 in the PA without recurrence group and control group

**Model**	**Genotype/allele**	**OR (95 % CI)**	***p*-value**	**AIC**
TAS2R16 rs860170				
Additive	C	0.740 (0.459–1.192)	0.216	394.021
Codominant	CT *vs.*TT<br>CC *vs.*TT	0.769 (0.472–1.252)	0.291	394.968
Dominant	CT+CC *vs.*TT	0.758 (0.466–1.234)	0.266	394.317
Overdominant	CT *vs.* TT+CC	0.789 (0.485–1.283)	0.339	394.640
Recessive	CC *vs.* CT+TT	–	–	–
TAS2R16 rs978739				
Additive	C	1.232 (0.842–1.804)	0.283	394.404

Codominant	CT *vs.*TT<br>CC *vs.*TT	1.482 (0.904–2.430)<br>1.070 (0.391–2.923)	0.119<br>0.896	395.070
Dominant	CT+CC *vs.*TT	1.419 (0.880–2.287)	0.151	393.481
Overdominant	CT *vs.* TT+CC	1.471 (0.909–2.380)	0.116	393.087
Recessive	CC *vs.* CT+TT	0.896 (0.337–2.382)	0.825	395.501
TAS2R16 rs1357949				
Additive	G	1.014 (0.707–1.455)	0.939	395.545
Codominant	AG *vs.* AA<br>GG *vs.* AA	0.909 (0.548–1.509)<br>1.143 (0.520–2.512)	0.713<br>0.740	397.184
Dominant	AG+GG *vs.* AA	0.951 (0.588–1.539)	0.838	395.508
Overdominant	AG *vs.* AA+GG	0.884 (0.548–1.425)	0.613	395.294
Recessive	GG *vs.* AA+AG	1.201 (0.572–2.524)	0.628	395.320

OR: odds ratio; CI: confidence interval; AIC: Akaike information criterion; *p*-value: significance level (statistically significant when $p < 0.017$).

Table S8. Binomial logistic regression analysis of TAS2R16 rs860170, rs978739, and rs1357949 in the PA with recurrence group and control group

**Model**	**Genotype/allele**	**OR (95 % CI)**	***p*-value**	**AIC**
TAS2R16 rs860170				
Additive	C	0.509 (0.246–1.052)	0.068	194.874
Codominant	CT *vs.*TT<br>CC *vs.*TT	0.521 (0.249–1.088)	0.082	196.649
Dominant	CT+CC *vs.*TT	0.513 (0.246–1.072)	0.076	195.076
Overdominant	CT *vs.* TT+CC	0.534 (0.256–1.114)	0.095	195.428
Recessive	CC *vs.* CT+TT			
TAS2R16 rs978739				
Additive	C	1.010 (0.563–1.810)	0.974	198.207
Codominant	CT *vs.*TT<br>CC *vs.*TT	0.858 (0.388–1.896)<br>1.295 (0.342–4.897)	0.705<br>0.704	199.847
Dominant	CT+CC *vs.*TT	0.925 (0.441–1.938)	0.836	198.165
Overdominant	CT *vs.* TT+CC	0.831 (0.384–1.801)	0.640	197.986
Recessive	CC *vs.* CT+TT	1.373 (0.375–5.026)	0.632	197.992
TAS2R16 rs1357949				
Additive	G	1.173 (0.677–2.032)	0.570	197.888
Codominant	AG *vs.* AA<br>GG *vs.* AA	1.001 (0.453–2.214)<br>1.538 (0.498–4.753)	0.997<br>0.454	199.601
Dominant	AG+GG *vs.* AA	1.097 (0.519–2.318)	0.808	198.149
Overdominant	AG *vs.* AA+GG	0.904 (0.434–1.884)	0.788	198.136
Recessive	GG *vs.* AA+AG	1.537 (0.541–4.371)	0.420	197.601

OR: odds ratio; CI: confidence interval; AIC: Akaike information criterion; *p*-value: significance level (statistically significant when $p < 0.017$).

Table S9. Distributions of TAS2R16 rs860170, rs978739, rs1357949 genotypes, and alleles in patients with PA and control group by PA activeness

**Genotype/ allele**	**Control group, n (%) (n=221)**	**Active PA group, n (%) (n=75)**	***p*-value**	**Not active PA group, n (%) (n=56)**	***p*-value**
TAS2R16 rs860170					
TT	78 (35.3)	33 (44.0)	0.307	25 (44.6)	0.355
CT	141 (63.8)	42 (56.0)		31 (55.4)	
CC	2 (0.9)	0 (0)		0 (0)	
T	297 (67.2)	108 (72.0)	0.274	81 (72.3)	0.298
C	145 (32.8)	42 (28.0)		31 (27.7)	
TAS2R16 rs978739					
TT	123 (55.7)	38 (50.7)	0.728	27 (48.2)	0.594
CT	83 (37.6)	32 (42.7)		25 (44.6)	
CC	15 (6.8)	5 (6.7)		4 (7.1)	
T	329 (74.4)	108 (72.0)	0.558	79 (70.5)	0.403
C	113 (25.6)	42 (28.0)		33 (29.5)	
TAS2R16 rs1357949					
AA	92 (41.6)	28 (37.3)	0.724	27 (48.2)	0.106
AG	106 (48.0)	40 (53.3)		19 (33.9)	
GG	23 (10.4)	7 (9.3)		10 (17.9)	
A	290 (65.6)	96 (64.0)	0.720	73 (65.2)	0.931
G	152 (34.4)	54 (36.0)		39 (34.8)	

p-value: significance level (statistically significant when $p < 0.017$).

Table S10. Binomial logistic regression analysis of TAS2R16 rs860170, rs978739, and rs1357949 in the not active PA group and control group

**Model**	**Genotype/allele**	**OR (95 % CI)**	***p*-value**	**AIC**
TAS2R16 rs860170				
Additive	C	0.664 (0.370–1.190)	0.169	278.992
Codominant	CT *vs.*TT<br>CC *vs.*TT	0.686 (0.378–1.244)	0.214	280.444
Dominant	CT+CC *vs.*TT	0.676 (0.373–1.226)	0.198	279.234
Overdominant	CT *vs.* TT+CC	0.704 (0.388–1.274)	0.246	279.544
Recessive	CC *vs.* CT+TT			
TAS2R16 rs978739				
Additive	C	1.218 (0.768–1.931)	0.403	280.187
Codominant	CT *vs.*TT<br>CC *vs.*TT	1.372 (0.745–2.528)<br>1.215 (0.374–3.949)	0.310<br>0.746	279.844
Dominant	CT+CC *vs.*TT	1.348 (0.749–2.426)	0.319	279.885
Overdominant	CT *vs.* TT+CC	1.341 (0.741–2.426)	0.332	279.946
Recessive	CC *vs.* CT+TT	1.056 (0.336–3.317)	0.925	280.870
TAS2R16 rs1357949				

Additive	G	1.019 (0.660–1.575)	0.932	280.871
Codominant	AG *vs.* AA<br>GG *vs.* AA	0.611 (0.319–1.170)<br>1.481 (0.629–3.492)	0.137<br>0.369	278.473
Dominant	AG+GG *vs.* AA	0.766 (0.425–1.380)	0.375	280.092
Overdominant	AG *vs.* AA+GG	0.557 (0.302–1.028)	0.061	277.258
Recessive	GG *vs.* AA+AG	1.871 (0.834–4.202)	0.129	278.714

OR: odds ratio; CI: confidence interval; AIC: Akaike information criterion; *p*-value: significance level (statistically significant when $p < 0.017$).

Table S11. Binomial logistic regression analysis of TAS2R16 rs860170, rs978739, and rs1357949 in the active PA group and control group

**Model**	**Genotype/allele**	**OR (95 % CI)**	***p*-value**	**AIC**
TAS2R16 rs860170				
Additive	C	0.680 (0.403–1.147)	0.148	334.999
Codominant	CT *vs.*TT<br>CC *vs.*TT	0.704 (0.413–1.200)	0.197	336.257
Dominant	CT+CC *vs.*TT	0.694 (0.407–1.183)	0.179	335.293
Overdominant	CT *vs.* TT+CC	0.722 (0.424–1.229)	0.230	335.653
Recessive	CC *vs.* CT+TT			
TAS2R16 rs978739				
Additive	C	1.133 (0.747–1.719)	0.557	336.740
Codominant	CT *vs.*TT<br>CC *vs.*TT	1.248 (0.723–2.155)<br>1.079 (0.368–3.163)	0.427<br>0.890	338.451
Dominant	CT+CC *vs.*TT	1.222 (0.723–2.065)	0.454	336.521
Overdominant	CT *vs.* TT+CC	1.237 (0.727–2.107)	0.433	336.470
Recessive	CC *vs.* CT+TT	0.981 (0.344–2.797)	0.971	337.080
TAS2R16 rs1357949				
Additive	G	1.081 (0.720–1.620)	0.708	336.942
Codominant	AG *vs.* AA<br>GG *vs.* AA	1.240 (0.710–2.160)<br>1.000 (0.388–2.575)	0.450<br>1.000	338.436
Dominant	AG+GG *vs.* AA	1.197 (0.698–2.052)	0.513	336.650
Overdominant	AG *vs.* AA+GG	1.240 (0.734–2.096)	0.422	336.436
Recessive	GG *vs.* AA+AG	0.886 (0.364–2.157)	0.790	337.010

OR: odds ratio; CI: confidence interval; AIC: Akaike information criterion; *p*-value: significance level (statistically significant when $p < 0.017$).

Table S12. Binomial logistic regression analysis of TAS2R16 rs860170, rs978739, and rs1357949 in the invasive PA group and control group

**Model**	**Genotype/allele**	**OR (95 % CI)**	***p*-value**	**AIC**
***TAS2R16* rs860170**				
Additive	C	0.874 (0.506–1.508)	0.627	320.002
Codominant	CT *vs.*TT	0.915 (0.523–1.602)	0.756	321.049

	CC *vs.*TT			
Dominant	CT+CC *vs.*TT	0.902 (0.515–1.579)	0.718	320.107
Overdominant	CT *vs.* TT+CC	0.938 (0.537–1.641)	0.823	320.187
Recessive	CC *vs.* CT+TT			
TAS2R16 rs978739				
Additive	C	0.874 (0.553–1.383)	0.566	319.903
Codominant	CT *vs.*TT<br>CC *vs.*TT	1.170 (0.672–2.035)<br>0.216 (0.028–1.687)	0.579<br>0.144	318.230
Dominant	CT+CC *vs.*TT	1.024 (0.595–1.763)	0.932	320.229
Overdominant	CT *vs.* TT+CC	1.279 (0.739–2.213)	0.379	319.467
Recessive	CC *vs.* CT+TT	0.202 (0.026–1.557)	0.125	316.538
TAS2R16 rs1357949				
Additive	G	0.985 (0.654–1.484)	0.942	320.231
Codominant	AG *vs.* AA<br>GG *vs.* AA	0.812 (0.455–1.448)<br>1.161 (0.486–2.776)	0.480<br>0.737	321.377
Dominant	AG+GG *vs.* AA	0.874 (0.507–1.507)	0.629	320.003
Overdominant	AG *vs.* AA+GG	0.787 (0.456–1.358)	0.389	319.489
Recessive	GG *vs.* AA+AG	1.291 (0.567–2.941)	0.543	319.876

OR: odds ratio; CI: confidence interval; AIC: Akaike information criterion; *p*-value: significance level (statistically significant when $p < 0.017$).

Table S13. Haplotype association of TAS2R16 rs860170, rs978739, rs1357949 with the predisposition to PA development

	**rs860170**	**rs978739**	**rs1357949**	**Frequency**	**OR (95 % CI)**	***p*-value**
1	T	T	G	0.334	1.00	
2	C	T	A	0.303	0.73 (0.45–1.18)	0.20
3	T	C	A	0.252	1.10 (0.74–1.64)	0.64
4	T	T	A	0.093	1.07 (0.61–1.88)	0.81
5	T	C	G	0.011	1.26 (0.24–6.60)	0.78
rare	*	*	*	0.006	0.00	1
Global haplotype association p–value: 0.5						

OR: odds ratio; CI: confidence interval; *p*-value: significance level (statistically significant when $p < 0.017$).
